# Fetal Atrial Flutter Associated with Atrial Septal Aneurysm

**DOI:** 10.3390/diagnostics12071722

**Published:** 2022-07-15

**Authors:** Fuanglada Tongprasert, Suchaya Luewan, Kasemsri Srisupundit, Theera Tongsong

**Affiliations:** Department of Obstetrics and Gynecology, Faculty of Medicine, Chiang Mai University, Chiang Mai 50200, Thailand; jeab094@hotmail.com (F.T.); suchaya.l@cmu.ac.th (S.L.); kasemsri.s@cmu.ac.th (K.S.)

**Keywords:** atrial flutter, atrial septal aneurysm, hydrops fetalis, premature atrial contraction

## Abstract

***Objective:*** To provide evidence that fetal atrial flutter (AF) caused by atrial septal aneurysm (ASA) can be completely cured by delivery. ***Methods:*** Cases series of three fetuses with ASA complicated by AF in late gestation, including hydrops fetalis in one case, were collected and completely followed up. ***Results:*** AF in all cases completely disappeared shortly after birth. New insights gained from this study are as follows: (1) PACs or bigeminy associated with ASA can progressively change to AF. (2) AF associated with ASA can cause hydrops fetalis and intrauterine treatment is needed; however, delivery is the definitive treatment. (3) AF associated with ASA completely resolves after birth. This is probably associated with changes in the circulation after birth, with no more blood flow crossing the foramen ovale and no turbulent flow in the ASA with reversal to hit the right atrial wall, activating ectopic pacemakers. ***Conclusions:*** This report may have clinical impact because it provides evidence that (1) in case of AF associated with ASA, the prognosis is much better than other causes and delivery should be strongly considered. (2) Fetuses diagnosed with AF should always be checked for the presence of ASA. (3) PAC/bigeminy related to ASA, different from isolated PAC, needs close follow-up for the development of SVT and AF. (4) Fetuses remote from term can benefit from intrauterine treatment to avoid hydrops fetalis, and to prolong gestation for maturity, early delivery is recommended once lung maturity is confirmed.

## 1. Introduction

Abnormal fetal heart rhythms are common, accounting for 1–2% of pregnancies. Irregular rhythm is one of the common reasons for referrals for fetal echocardiography. The majority of cases are benign atrial ectopic beats, whereas sustained fetal bradyarrhythmias or tachyarrhythmias, which are associated with significant neonatal morbidity and mortality, account for less than 10% of referrals [1]. Supraventricular tachycardia (SVT), a rate range of approximately 220–240 bpm with a 1:1 ratio of AV conduction, is the most common cause of fetal tachyarrhythmia, accounting for 66–90% of all cases [2]. SVT is typically caused by an accessory pathway between the atrium and the ventricle, which allows retrograde reentry of VA conduction. Atrial flutter (AF) is a form of SVT involving accessory circuits within the atria, causing atrial reentrant tachycardia. AF is typically associated with an atrial rate of 300–600 bpm with variable degrees of AV conduction block, resulting in a slower ventricular rate of 220–240 bpm, accounting for 10–30% of fetal tachyarrhythmias [2] and associated with hydrops fetalis in 35–40% [3].

Atrial septal aneurysm (ASA) was first described in 1966 by Thompson et al. [4]. Early in the fetal development of the interatrial septum, a thin membranous septum primum is firstly created in the fetal atrium to separate the atria into the left and the right. Shortly after that, the thicker septum secundum develops in the right atrium close to the septum primum and grows alongside the septum primum. In the case that the septum primum tissue grows extensively or its supporting tissue is scarce, the loose and wide septum primum flap becomes excessive and mobile [5]. If the enlarged septum primum flap or the flap of the foramen ovale in intrauterine life excessively extends into the left atrium, this tissue flap is defined as atrial septal aneurysm (ASA) or a redundant septum primum flap or foramen ovale aneurysm [6]. ASA in newborns, defined as an excursion ratio (maximal excursion of the septum primum to left atrial diameter ratio) of 25% or greater, is common in newborns, with possibly as many as 7.6% [7], and all cases disappear at the end of the first year of life, mostly in one month, without any complications related to the lesion during the follow-up period [7]. Several cases of prenatal diagnosis of atrial septal aneurysms have been reported [8,9,10,11,12,13,14,15,16], with most of them resulting in good neonatal outcomes with serious consequences. Thus, ASA can be considered as a benign and transient observation. Nevertheless, ASA associated with dysrhythmias in newborns or adults has been recognized [17,18,19,20]. ASAs are occasionally observed as an incidental finding that is typically benign. However, they may be associated with more significant pathological changes such as mitral valve prolapse and various other malformations [21,22]. ASA in fetuses should be defined differently, since an excursion ratio of 25% can be a physiologic movement caused by blood flow crossing the foramen ovale. In fetuses, the cut-off point for diagnosis of excessive excursion is defined as greater than 1.3 MoM or an excursion ratio of greater than 55% [23], whereas some authors use a cut-off of 50% [6]. As mentioned earlier, ASA may be associated with dysrhythmias, especially, premature atrial contractions (PACs), which is the most common type of arrhythmias reported to be secondary to ASA [10,24]. Fetal PACs usually have a good prognosis and disappear spontaneously during pregnancy or shortly after birth [25]. PACs may rarely develop secondarily to underlying structural abnormalities. The presence of ASA is the most commonly reported structural anomaly in fetuses [26,27]. However, atrial flutter (AF) secondary to ASA in fetuses is rarely described prenatally. AF is seen in about 30% of fetuses with tachyarrhythmia [28]. Most fetuses with prenatal AF have an accessory AV connection and can develop reentrant SVT in utero or after birth. The atrial rate for AF is usually 300–500 beats/min and is usually sustained with variable AV block, whereas the atrial rate for other atrial tachycardias is either nonsustained or sustained at an atrial rate of 180–240 beats/min. The ventricles cannot respond in a 1:1 fashion to the extremely fast atrial rates and so there is 2:1 or variable atrioventricular block [29,30]. Most cases of fetal AF are not associated with cardiac anomalies. In one large retrospective study, not a single case of ASA was identified among 44 fetuses with AF [31]. To the best of our knowledge, fetal ASA associated with AF has never been reported, though supraventricular tachycardia associated with ASA was published in older patients [20]. The objective of this study was to describe cases of fetal ASA complicated by AF in late gestation, including hydrops fetalis in one case, with complete disappearance shortly after birth, to underline the clinical impact of AF secondary to ASA and to emphasize the close follow-up of fetuses with PAC associated with ASA.

## 2. Case Series

This case series was carried out with ethical approval by the Institutional Review Boards (IRB) (Faculty of Medicine, Chiang Mai University) and the patients provided written informed consent.

**Figure 1:**
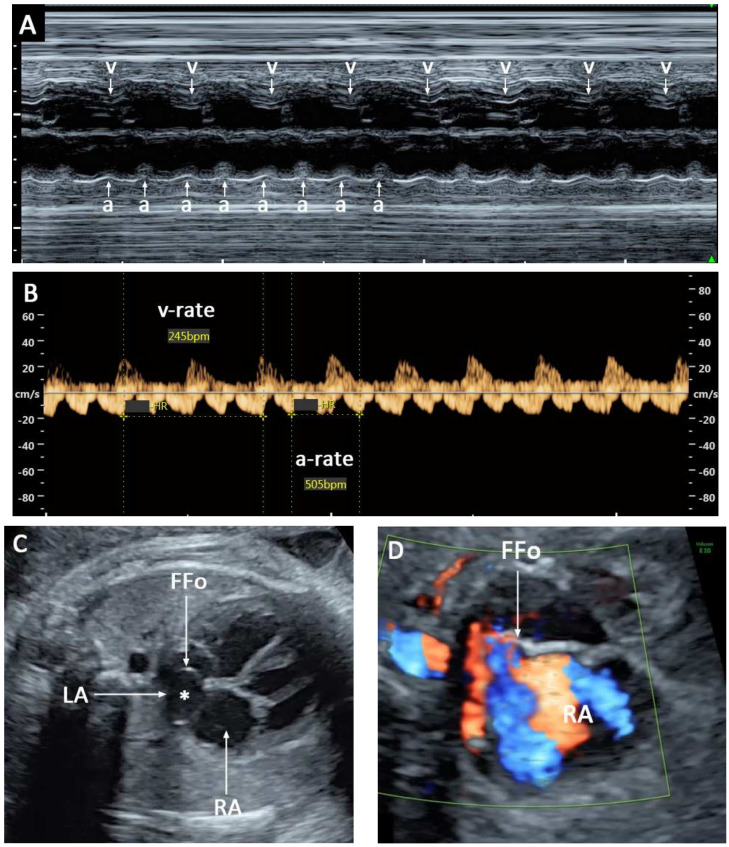
(**A**) M-mode shows a ventricular rate of (v) 245 bpm and an atrial rate of (a) 490 bpm; (**B**) Doppler waveforms of the renal vein (a-rate: 245 bpm) and renal artery (v-rate: 505 bpm); (**C**): Atrial septal aneurysm (*): wide excursion of the foramen ovale flap; (**D**): Color flow mapping in the septal aneurysm shows turbulent reversed flow returning back to the right atrium (FFo: flap of foramen ovale; LA: left atrium; RA: right atrium).

**Case 1:** A 33-year-old pregnant woman, G2 P1001, attended the scheduled antenatal care at 35 weeks of gestation at a community hospital. On physical examination, fetal tachycardia with a heart rate greater than 200 bpm was detected. The pregnancy was referred to our center for further management. Her history of the prenatal course was uneventful. Fetal anomaly screening at 20 weeks of gestation was normal and the chromosome study result was low risk for aneuploidy. At our center, at 35 weeks, detailed ultrasound showed a normally formed female fetus with normal growth and fetal biometry, occasional PAC, alternated with occasional atrial flutter (AF) (Figure 1A,B) and with normal rhythm. However, the fetal heart had atrial septal aneurysm (ASA), with an abnormally wide excursion of the foramen ovale flap (approximately 80% of left atrial diameter: Figure 1C) with turbulent reversed flow in the septum back to the right atrium (Figure 1D), instead of crossing the foramen.

The patient was admitted for close follow-up with ultrasound and expectantly treated. The dysrhythmias were more progressive, turning out to be persistent AF. During surveillance, at 36^+5^ weeks of gestation, ultrasound showed that the fetal atrial rate was 505 bpm and the ventricular rate was 245 bpm. AF with 2:1 AV block was diagnosed. AF was persistent after observation over a period of four hours. The cardiac size was within the normal limit. No hydropic sign was detected. The last fetal heart rate 2 weeks before this admission was normal (150 bpm), as recorded by Doppler device.

Because the AF was persistent, without an alternate normal rhythm, early treatment was needed. Since the gestational age was nearly term, rather than in utero treatment, cesarean delivery was performed for early treatment after birth. The female newborn had a birthweight of 2750 g, no gross structural anomaly, and an Apgar score of 10 at 5 min. Immediately after birth, the heart rate was first detected to be approximately 240 bpm. Ten minutes later, the heart rate was suddenly reduced to 124 bpm, no longer tachycardia. On neonatal echocardiography, the heart was otherwise normal except the small size of the atrial septal aneurysm with a minimal redundant atrial septum, bulging predominantly into the right atrium, with no significant turbulent flow in the septum as previously shown in utero. The AF completely disappeared without recurrence.

**Figure 2:**
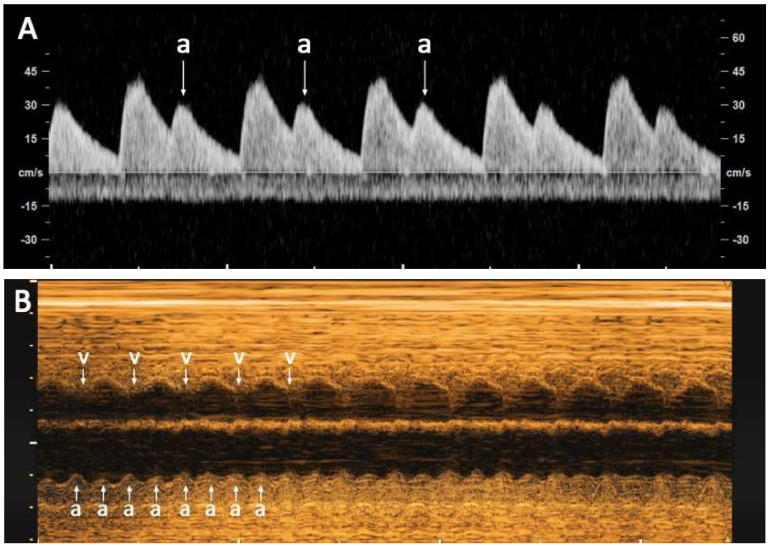
(**A**) Umbilical artery waveforms show premature atrial contraction/bigeminy (a): (**B**) M-mode shows atrial flutter; atrial rate (a): ventricular rate (v) 2:1).

**Case 2:** A 20-year-old pregnant woman, G0 P0, attended routine antenatal care at 33^+4^ weeks of gestation at a private hospital. On physical examination, fetal tachycardia with a heart rate of approximately 230 bpm was detected. The pregnancy was referred to our center because of fetal tachycardia. The pregnancy was low risk and the prenatal course was unremarkable. Fetal anomaly screening and fetal biometry at 21 weeks of gestation was normal. Fetal Down syndrome screening was low risk. At our center, the fetal heart rate was sometimes irregular and sometimes very fast (approximately 240 bpm). The detailed ultrasound examination at 33^+4^ weeks revealed a normally formed male fetus with appropriate growth. Nevertheless, fetal tachycardia was observed. The fetal heart had tortious ductus arteriosus and ASA (abnormally wide excursion of the foramen ovale flap: approximately 80% of left atrial diameter) with turbulent reversed flow in the septum, circulating back to the right atrium. The fetal atrial rate was varied, occasional bigeminy of PAC with conduction with a rate of about 150 bpm (Figure 2A), with an occasional normal rate of 140–150 bpm, but mostly tachycardia with an atrial rate of 480 bpm and the ventricular rate was 240 bpm (Figure 2B). AF with 2:1 AV block was diagnosed, and the AF took most of the time. Because AF was not persistent and there were no hydropic signs, other than mild cardiomegaly (cardio-thoracic diameter ratio of 65%), expectant management with close follow-up was given. Fetal well-being was assessed by the biophysical profile. At 36 weeks of gestation, AF was persistent. We decided to perform a cesarean delivery for postnatal treatment, giving birth to a 2540-g male newborn, with an Apgar score of 10 at 5 min. Immediately after the birth, the heart rate was regular, 120 bpm, no longer tachycardia. Neonatal echocardiography revealed normal cardiac structures except the atrial septal aneurysm, with a redundant atrial septum and no flow crossing the foramen ovale. The AF and hydropic signs completely disappeared without any treatment.

**Figure 3:**
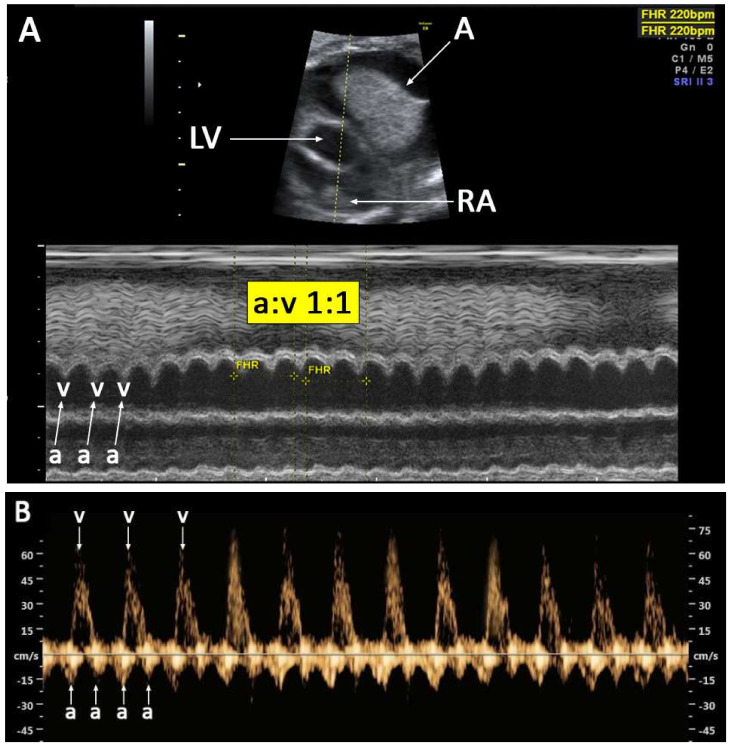
(**A**) M-mode shows supraventricular tachycardia; atrial rate (a): ventricular rate (v) (A): ascites; LA: left atrium; RA: right atrium); (**B**) cardiac Doppler waveforms at left inflow and outflow shows atrial flutter; atrial rate (a): ventricular rate (v) 2:1 (440:226).

**Case 3:** A 25-year-old pregnant woman, G2 P1001, attended our antenatal care at 37^+4^ weeks of gestation, with incidental findings of atrial septal aneurysm with fetal tachycardia, and a heart rate of approximately 220 bpm. She attended her first antenatal visit at 12^+4^ weeks of gestation. The antenatal course was low risk and uneventful. Fetal anomaly screening at 20^+5^ weeks of gestation was unremarkable. The second trimester serum screening was low risk. At 33^+4^ weeks of gestation, the fetus developed occasional PACs with conduction and ASA was first noted, with a maximal excursion of about 80% of the left atrial width. The fetal heart rate was relatively normal, though sometime irregular. Expectant management and close follow-up as out-patient care was provided. At 34^+4^ weeks, the fetus developed persistent SVT, with an atrial rate to ventricular rate ratio of 1:1 (220 bpm) (Figure 3A). Additionally, hydropic signs, including pleural effusion and ascites, were observed. Because SVT was persistent during the preterm period, intrauterine treatment with flecainide via maternal circulation was instituted. Drug administration and the dosage of flecainide are described elsewhere [31]. Approximately 24 h after treatment, cardioversion to a normal sinus rhythm was successful. However, four days after the initiation of intrauterine treatment, AF developed despite intrauterine treatment (Figure 3B). Then, the combination of digoxin (0.5 mg/d) and flecainide was administered instead. Nevertheless, cardioversion was not successful. We decided to perform delivery as early postnatal treatment. Cesarean delivery was performed at 35^+6^ weeks of gestation, giving birth to a normally formed male newborn, with a birthweight of 2550 g and an Apgar score of 9 at 5 min. The neonatal heart (ventricular) rate in the delivery room was 240 bpm. Shortly after that, at the NICU, the heart rate suddenly lowered to 130 bpm, with no need for any anti-arrhythmic medication. Neonatal echocardiography showed a marked decrease in the maximal excursion of the foramen ovale flap, with no significant turbulent flow in the septum as previously shown in utero. There was a trivial low-velocity left to right shunt across the patent foramen ovale. The AF and hydropic signs completely disappeared without recurrence.

A summary of the characteristics of the three cases with AF associated with ASA is presented in Table 1.

## 3. Discussion

New insights gained from this study are as follows: (1) Though PACs or bigeminy are usually benign, those associated with ASA have a tendency to subsequently develop into SVT or AF. Hypothetically, the dysrhythmias may be activated by mechanical stimulation of turbulent flow produced by ASA. (2) AF associated with ASA has a tendency to develop in late pregnancy. (3) AF associated with ASA can cause hydrops fetalis and intrauterine treatment is needed, especially in the case of being remote from term, to prolong gestation for maturity. However, delivery is the definitive treatment. (4) AF associated with ASA has a good prognosis and usually resolves shortly after birth. This is probably associated with changes in the circulation after birth, with no more blood flow across the foramen ovale and no turbulent flow in the septum with reversal to hit the right atrial wall.

The mechanism of the development of fetal arrhythmias or atrial flutter caused by ASA is unclear. Some hypotheses have been proposed as follows. Papa et al. reported ASA in 93 of 1223 fetuses (7.6%), with 36% of them being associated with PACs [27]. They proposed that the pressure exerted by the redundant septum primum on the atria caused the ectopic rhythm. In one earlier study, Rice et al. reported that there was a statistically significant association between the diagnosis of fetal atrial tachyarrhythmias and an ASA, which was demonstrated by echocardiography. Additionally, they hypothesized that ASA contact with the left atrial free wall or mitral valve annulus could be the nidus for the arrhythmia. Nevertheless, postnatal work-up to confirm the prenatal findings was not performed [10]. Later, Morales et al. [32] provided direct echocardiographic evidence of a previously proposed pathogenesis for the development of an atrial arrhythmia caused by ASA through direct contact with the left atrial posterior wall. The authors captured the ASA extruding into the left atrium, which leads to a PAC. The authors also documented resolution of the arrhythmia with conservative efforts in newborns with an ASA because of normal physiologic adaptation associated with the extra-uterine life transition. Immediately after delivery, normalized lung circulation facilitates the pulmonary venous return and the left atrial pressure to increase and, therefore, the pressure difference between the right and left atria becomes prominent. As a result of this increased pressure difference between the atria, the flap of the foramen ovale adheres and is fixed to the septum secundum and its movement is limited, eliminating the cause of the PACs [14].

Though AF is usually associated with high morbidity and mortality, we have encountered three cases of AF with good prognosis, which completely resolved by delivery, and all of them had septal aneurysms. Thus, it is reasonable to assume that AF is likely caused by ASA, since delivery, which resulted in dramatic change in fetal to neonatal hemodynamics with functional close of the foramen ovale or no more blood flow crossing the foramen, resulted in AF disappearing. In other words, delivery gets rid of turbulent reversed flow, which could interfere with atrial ectopic pacemakers, leading to the disappearance of AF. Accordingly, delivery is a good option in cases of near-term pregnancies. This is also a valid option for fetuses with AF not associated with ASA, since postnatal treatment of tachyarrhythmia is usually effective. There is little reason to pursue transplacental drug therapy for the term fetus with tachyarrhythmia but no hydrops [33].

Of interest, although the number of cases is very small, the three cases strongly suggest that ASA is likely associated with SA node dysfunction or activation of an ectopic foci in the right atrial wall. We hypothesize that the turbulent flow in the aneurysms, circulating back to the right atrium instead of crossing the foramen ovale to the left atrium, may activate the ectopic pacemaker foci to initiate dysrhythmias. Fetal ASA may be associated with autonomic dysfunction, as reported in adult patients [34]. However, the natural course of AF secondary to excessive septal excursion in the fetus can be reversed by delivery, which is different from the persistent form seen in adult. Because delivery gets rid of the abnormal flow, which is the definitive treatment, the prognosis of AF associated with ASA is very good.

Typically, medical treatment might not be required for neonatal atrial arrhythmias (PACs in most cases) caused by ASA as long as the newborns remain hemodynamically stable. Expectant management during the natural transition from intra-uterine to extra-uterine life and normalization of the ASA redundancy may be sufficient. Nevertheless, each case should be managed on an individual basis and based on the clinical status. Based on our observations, AF, usually associated more with neonatal morbidity than PACs, always requires either intrauterine or extra-uterine treatment; however, when it is a consequence of ASA, AF is likely to spontaneously disappear after birth, when physiologic adaptation to an equal redistribution of blood flow in both atria occurs in newborns. Our findings suggest that ASA-associated AF abates with resolution of the ASA in extra-uterine adaptation. Notably, Martucci et al. [35] reported a fetus with pseudobradycardia caused by trigeminal PACs at week 33 of pregnancy. Their case also showed a normal rhythm immediately after birth. This finding is consistent with our findings, despite different types of arrhythmias, supporting the mechanism that extra-uterine normalization facilitates the resolution of arrhythmias. However, contrary to our cases, the case of Martucci et al. developed trigeminal PACs and supraventricular tachycardia (SVT) on day 11 of neonatal life and needed treatment. Therefore, though the arrhythmias associated with ASA usually disappear shortly after birth, close monitoring of newborns is still necessary.

**Clinical impact:** We provide evidence that fetuses diagnosed with AF should always be carefully checked for the presence of ASA. Different from isolated PAC or bigeminy, PAC or bigeminy associated with ASA needs close follow-up for the development of SVT and AF. Early detection of AF and early intrauterine treatment is very critical, since undiagnosed and untreated AF can result in fetal heart failure, hydrops fetalis, and dead fetus in utero. In case of atrial flutter associated with ASA, the prognosis is much better than other causes and delivery should be strongly considered. Fetuses remote from term can benefit from intrauterine treatment to avoid hydrops fetalis, and to prolong gestation for maturity, early delivery is recommended once lung maturity is confirmed.

## 4. Conclusions

AF can be caused by ASA and has a tendency to develop in late pregnancy. It may be preceded by PAC or bigeminy or supraventricular tachycardia. Hypothetically, turbulent flow in the aneurysms returning to the right atrium may mechanically activate ectopic pacemaker foci or SA node dysfunction. Different from most cases of AF, spontaneous disappearance can be expected after delivery. However, untreated AF associated with ASA can cause hydrops fetalis and intrauterine treatment should be instituted. In practice, when fetal arrhythmias are detected, ASA should be listed in differential diagnoses and carefully looked for. In cases of ASA-associated arrhythmias, fetuses should have a serial ultrasound examination every 1–2 weeks for the development of SVT or AF and early signs of hydrops fetalis for early intrauterine treatment or referral to a tertiary care center.

## Figures and Tables

**Table 1:** Characteristics of the three cases with atrial flutter associated with septal aneurysms.

Case No.	Maternal Age	GA at Diagnosis (Week)	Intrauterine Treatment	GA at Delivery (Week)	Hydrops Fetalis	Preceding Arrhythmia	Birth Weight
1	33	35	No	36^+5^	No	Bigeminy	2750
2	20	33^+4^	No	36	No	PACs, Bigeminy	2540
3	25	34^+4^	Yes	35^+6^	Yes	PACs, SVT	2550

## Data Availability

The data of this report are available from the corresponding authors upon request.

## References

[1] Reed K.L. (1989). Fetal arrhythmias: Etiology, diagnosis, pathophysiology, and treatment. Semin. Perinatol..

[2] Van Engelen A.D., Weijtens O., Brenner J.I., Kleinman C.S., Copel J.A., Stoutenbeek P., Meijboom E.J. (1994). Management outcome and follow-up of fetal tachycardia. J. Am. Coll. Cardiol..

[3] Krapp M., Kohl T., Simpson J.M., Sharland G.K., Katalinic A., Gembruch U. (2003). Review of diagnosis, treatment, and outcome of fetal atrial flutter compared with supraventricular tachycardia. Heart.

[4] Thompson J.I., Phillips L.A., Melmon K.L. (1966). Pseudotumor of the right atrium. Report of a case and review of its etiology. Ann. Intern. Med..

[5] Stewart P.A., Wladimiroff J.W. (1988). Fetal atrial arrhythmias associated with redundancy/aneurysm of the foramen ovale. J. Clin. Ultrasound JCU.

[6] Kachalia P., Bowie J.D., Adams D.B., Carroll B.A. (1991). In utero sonographic appearance of the atrial septum primum and septum secundum. J. Ultrasound Med..

[7] Ozcelik N., Atalay S., Tutar E., Ekici F. (2006). Prevalence of interatrial septal aneurysm in newborns and their natural course. Pediatr. Cardiol..

[8] Haddad S., Degani S., Rahav D., Ohel G. (1996). The antenatal diagnosis of fetal atrial septal aneurysm. Gynecol. Obs. Investig..

[9] Pinette M.G., Pan Y., Pinette S.G., Blackstone J., Stubblefield P.G. (1997). Fetal atrial septal aneurysm. Prenatal diagnosis by ultrasonography. J. Reprod. Med..

[10] Rice M.J., McDonald R.W., Reller M.D. (1988). Fetal atrial septal aneurysm: A cause of fetal atrial arrhythmias. J. Am. Coll. Cardiol..

[11] Santos A.C., Branco M., Martins P. (2020). Fetal atrial septal aneurysm: A differential diagnosis of aortic arch retrograde flow. BMJ Case Rep..

[12] Sun H.Y., Fripp R.R., Printz B.F. (2015). Unusual consequence of a fetal atrial septal aneurysm. Clin. Case Rep..

[13] Karmegaraj B. (2021). Prenatal diagnosis of redundant foramen ovale flap aneurysm prolapsing into mitral valve mimicking coarctation of aorta. Ultrasound Obs. Gynecol..

[14] Toro L., Weintraub R.G., Shiota T., Sahn D.J., Sahn C., McDonald R.W., Rice M.J., Hagen-Ansert S. (1994). Relation between persistent atrial arrhythmias and redundant septum primum flap (atrial septal aneurysm) in fetuses. Am. J. Cardiol..

[15] Vena F., Donarini G., Scala C., Tuo G., Paladini D. (2020). Redundancy of foramen ovale flap may mimic fetal aortic coarctation. Ultrasound Obs. Gynecol..

[16] Hung J.H., Lu J.H., Hung C.Y. (2008). Prenatal diagnosis of atrial septal aneurysm. J. Clin. Ultrasound JCU.

[17] Russo V., Rago A., Di Meo F., Papa A.A., Ciardiello C., Cristiano A., Calabrò R., Russo M.G., Nigro G. (2015). Atrial Septal Aneurysms and Supraventricular Arrhythmias: The Role of Atrial Electromechanical Delay. Echocardiography.

[18] Yetkin E., Ileri M., Korkmaz A., Ozturk S. (2020). Association between atrial septal aneurysm and arrhythmias. Scand. Cardiovasc. J. SCJ.

[19] Bozkaya V., Oskovi-Kaplan Z.A., Engin-Ustun Y. (2020). Atrial septal aneurysm in pregnancy: Echocardiography and obstetric outcomes. J. Perinat. Med..

[20] Hauser A.M., Timmis G.C., Stewart J.R., Ramos R.G., Gangadharan V., Westveer D.C., Gordon S. (1984). Aneurysm of the atrial septum as diagnosed by echocardiography: Analysis of 11 patients. Am. J. Cardiol..

[21] Mattioli A.V., Bonetti L., Aquilina M., Oldani A., Longhini C., Mattioli G. (2003). The association between atrial septal aneurysm and mitral valve prolapse in patients with recent stroke and normal carotid arteries. Ital. Heart J. Off. J. Ital. Fed. Cardiol..

[22] Sahn D.J., Allen H.D., Anderson R., Goldberg S.J. (1978). Echocardiographic diagnosis of atrial septal aneurysm in an infant with hypoplastic right heart syndrome. Chest.

[23] Sirilert S., Tongprasert F., Srisupundit K., Luewan S., Tongsong T. (2016). Fetal septum primum excursion (SPE) and septum prmum excursion index (SPEI) as sonomarkers of fetal anemia: Using hemoglobin Bart’s fetuses as a study model. Prenat. Diagn..

[24] Yozgat Y., Kilic A., Karadeniz C., Ozdemir R., Doksoz O., Mese T., Unal N. (2013). Importance of close follow-up in the fetus with premature atrial contractions accompanied by atrial septal aneurysm: A case report. Case Rep. Obstet. Gynecol..

[25] Copel J.A., Friedman A.H., Kleinman C.S. (1997). Management of fetal cardiac arrhythmias. Obs. Gynecol. Clin. N. Am..

[26] (2009). ACOG Practice Bulletin No. 106: Intrapartum fetal heart rate monitoring: Nomenclature, interpretation, and general management principles. Obs. Gynecol..

[27] Papa M., Fragasso G., Camesasca C., Di Turi R.P., Spagnolo D., Valsecchi L., Calori G., Margonato A. (2002). Prevalence and prognosis of atrial septal aneurysm in high risk fetuses without structural heart defects. Ital. Heart J. Off. J. Ital. Fed. Cardiol..

[28] Yuan S.M. (2020). Fetal arrhythmias: Diagnosis and treatment. J. Matern. Fetal. Neonatal. Med..

[29] Simpson J.M. (2006). Fetal arrhythmias. Ultrasound Obs. Gynecol..

[30] Yuan S.M., Xu Z.Y. (2020). Fetal arrhythmias: Prenatal evaluation and intrauterine therapeutics. Ital. J. Pediatr..

[31] Lisowski L.A., Verheijen P.M., Benatar A.A., Soyeur D.J., Stoutenbeek P., Brenner J.I., Kleinman C.S., Meijboom E.J. (2000). Atrial flutter in the perinatal age group: Diagnosis, management and outcome. J. Am. Coll. Cardiol..

[32] Morales R., Bokowski J.W., Nguyen H., Awad S.M. (2019). A Proposed Etiology for Atrial Tachyarrhythmia in Neonates with Atrial Septal Aneurysms. Pediatric. Cardiol..

[33] Veduta A., Panaitescu A.M., Ciobanu A.M., Neculcea D., Popescu M.R., Peltecu G., Cavoretto P. (2021). Treatment of Fetal Arrhythmias. J. Clin. Med..

[34] Demir M. (2013). The relationship between atrial septal aneurysm and autonomic dysfunction. Exp. Clin. Cardiol..

[35] Martucci V., Cerekja A., Caiaro A., Bosco G., Lucchini R., Piacentini G., Marino B., Ventriglia F. (2012). Blocked atrial bi/trigeminy in utero evolving in supraventricular tachycardia after birth. Case Rep. Obstet. Gynecol..

